# Surface-Enhanced Raman Spectroscopy for Cancer Immunotherapy Applications: Opportunities, Challenges, and Current Progress in Nanomaterial Strategies

**DOI:** 10.3390/nano10061145

**Published:** 2020-06-11

**Authors:** Shuvashis Dey, Matt Trau, Kevin M. Koo

**Affiliations:** 1 Centre for Personalized Nanomedicine, Australian Institute for Bioengineering and Nanotechnology (AIBN), The University of Queensland, Brisbane, QLD 4072, Australia; 2 School of Chemistry and Molecular Biosciences, the University of Queensland, Brisbane, QLD 4072, Australia; 3 XING Technologies Pty Ltd., Brisbane, QLD 4073, Australia; 4 The University of Queensland Centre for Clinical Research (UQCCR), Brisbane, QLD 4029, Australia

**Keywords:** surface-enhanced Raman spectroscopy, cancer immunotherapy, tumor microenvironment, multiplexed detection, nanomaterials

## Abstract

Cancer immunotherapy encompasses a variety of approaches which target or use a patient’s immune system components to eliminate cancer. Notably, the current use of immune checkpoint inhibitors to target immune checkpoint receptors such as CTLA-4 or PD-1 has led to remarkable treatment responses in a variety of cancers. To predict cancer patients’ immunotherapy responses effectively and efficiently, multiplexed immunoassays have been shown to be advantageous in sensing multiple immunomarkers of the tumor microenvironment simultaneously for patient stratification. Surface-enhanced Raman spectroscopy (SERS) is well-regarded for its capabilities in multiplexed bioassays and has been increasingly demonstrated in cancer immunotherapy applications in recent years. This review focuses on SERS-active nanomaterials in the modern literature which have shown promise for enabling cancer patient-tailored immunotherapies, including multiplexed in vitro and in vivo immunomarker sensing and imaging, as well as immunotherapy drug screening and delivery.

## 1. Introduction

**Figure 1:**
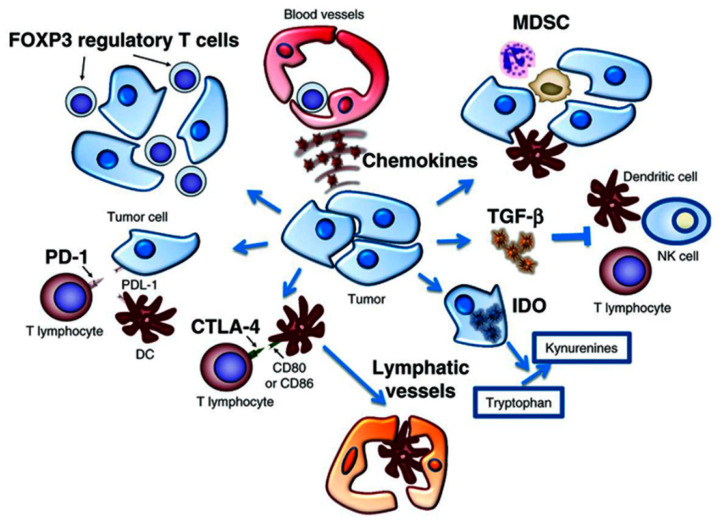
The biological cellular and molecular interactions within the complex tumor microenvironment. Solid tumor cells interact with a variety of cells and molecules, including lymphocytes, cytokines, chemokines, dendritic cells, and T cells, among others. The cells and molecules which create this tumor microenvironment can serve as excellent immunomarkers for various cancer immunotherapy applications. Reproduced from [7], with permission from the American Association for Cancer Research, 2012.

Cancer is a dynamic disease that can engage multiple immune evasion strategies to promote tumor growth and spread [1,2,3,4,5]. The tumor microenvironment is a complex system of immune cells, cytokines, chemokines, soluble factors, growth factors, and adhesion molecules which drives malignant tumor cell interactions with surrounding normal cells (Figure 1) [6,7,8,9]. Cancer immunotherapy is based on the reprogramming of molecular mechanisms that govern the interplay between cancer cells and immune cells within the tumor microenvironment. The tumor microenvironment is critical in cancer initiation and spread, as well as therapeutic responses and resistance. As such, recent landmark progress and ongoing efforts in cancer treatment of the last decade focus on targeting various components of the tumor microenvironment as cutting-edge cancer immunotherapies [10,11,12,13,14]. Widely utilized cancer immunotherapy approaches include immune checkpoint blockade therapy directed against immune checkpoint proteins (e.g., PD-1, PD-L1, CTLA-4, TIM-3, VISTA), chimeric antigen receptor T (CAR-T) cells, dendritic cell vaccines, and cytokines, among others [15,16,17,18,19,20]. However, therapy outcomes for the same immunotherapy vary from patient to patient due to the engagement of different immune evasion pathways. Therefore, it is essential to profile biomarkers within the tumor microenvironment for predicting the efficacy of immunotherapy among responders and non-responders [21,22,23,24,25,26,27]. 

One strategy to improve the efficacy of current cancer immunotherapies relies on the better identification and detection of molecular biomarkers within the tumor microenvironment to predict and monitor tumor response. For instance, the use of an anti-PD-L1 biomarker immunoassay on tumor tissues has been clinically validated and received FDA (Food and Drug Administration) approval for the prediction of response to first-line immunotherapy in certain cancer types [28,29]. It can thus be envisaged that with advances in immunomarker discoveries in solid tissue and/or liquid sample matrices, molecular signatures within the tumor microenvironment can guide the precise selection of cancer immunotherapies. Still, from a biosensing perspective, the task of immunomarker detection within the intricate yet interconnecting tumor microenvironment is extremely challenging. This is due to the need for simultaneous profiling of multiple biomarkers that may be present in trace quantities. 

In relation to these said challenges, recent progress in surface-enhanced Raman spectroscopy (SERS)-active nanomaterials to impart both excellent SERS immunomarker detection sensitivity and multiplexity has gained significant attention. In brief, SERS detection involves laser activation and signal acquisition on surface-modified metallic nanomaterials that are used for labeling target biomolecules via specific interactions [30,31,32]. SERS-active nanomaterials confer excellent Raman signal enhancement efficiency and can be encoded with various organic Raman reporter molecules for the multiplexed detection of various biomolecules via innovative quantification modes [33,34,35,36,37]. Thus, this combination of cutting-edge nanomaterials and spectroscopic effect has enabled the use of SERS in miniaturized reaction volumes with exceptional detection performance [38]. Typically, gold nanostructures are the most common nanomaterial for SERS immunoassays due to their biocompatibility and facile surface modification chemistries for targeting moieties, contrast agents, and/or stabilizing molecules. 

Although the general use of SERS-active nanomaterials has been extensively reviewed in recent times, a specific focus on the latest usage of SERS-active nanomaterials for decoding tumor–host immune interactions is yet to be summarized [30,33,39,40,41]. In a bid to provide a unique outline on the emerging use of SERS in the exciting field of cancer immunotherapy, we herein discuss the recent progress in SERS nanomaterials that have exhibited application potential for patient-tailored cancer immunotherapy selection and monitoring, including multiplexed in vitro and in vivo immune-sensing, -imaging, and -drug screening/delivery.

## 2. SERS Immunomarker Sensing on Cells and in Circulation

The success of cancer immunotherapeutic approaches is largely dependent on the efficient identification of a variety of immunomarkers in the tumor microenvironment, against which therapeutic approaches can be engaged for the best possible outcome [42,43,44]. SERS-active nanomaterials have been developed for SERS immunomarker sensing and immunotherapy stratification, with the latest reports including immune checkpoint blockade immunomarkers on the cell surface, as well as in circulation. The most common SERS-active nanomaterial-based strategy for immunomarker sensing involves the incubation of antibody-coated metallic SERS nanoparticles with target immunomarkers, followed by subsequent excitation with a laser source and SERS signal acquisition to quantitatively reflect the presence of target biomarkers in the sample. 

Stepula et al. reported an Au/Au core/satellite nanoparticles-based SERS assay technique for the specific and sensitive analysis of the PD-L1 biomarker expression on cancer cell surfaces [45]. Herein, the Raman signature of the reporter molecules attached on the antibody-functionalized Au/Au core/satellite nanoparticles significantly enhances the Raman signal strength, hence improving the detection sensitivity. This enhancement of the Raman signal is highly influenced by the plasmonic coupling between the Au core and the Au satellites of nanoparticles that leads to the formation of a very high local electric field, termed as hot spots, for Raman signal amplification [45]. 

The detection and characterization of CD8^+^ T cells, one of the key regulators for immunoregulation, has also been demonstrated with a composite organic–inorganic nanoparticles (COINs)-based SERS assay. COINs are a modified form of SERS nanoparticles with a significantly enhanced Raman signal, with around a 10^4-5^-fold higher intensity as compared with single silver nanoparticles. COINs are clusters of silver nanoparticles tagged with Raman dyes and prepared in the presence of heat or salt, and the increased aggregation of nanoparticles facilitate significantly enhanced Raman signals. Utilizing this method, Sailaja and co-workers successfully detected CD8^+^ T cells (as low as 7% of total cells) from a background of peripheral blood mononuclear cells. Furthermore, they also demonstrated the utility of COINs for intracellular phosphorylation signaling in a U937cell line model treated with IL (interleukin)-4 and IFN (interferon)-γ [46]. In another study showing the detection of immunomarkers on leukemic lymphocytes, Zhang et al. employed gold nanoparticles capped by Raman reporter molecules and antibodies, and then encapsulated by polyethylene glycol for specifically targeting the CD3 or CD19 expression on target cells [47].

Parallel to surface bound protein biomarkers, immunomarkers present in circulation are also gaining significant attention for their potential clinical applications in both disease diagnosis and treatment monitoring. Li et al. reported the use of gold–silver alloy nanoboxes as SERS-active nanomaterials for the simultaneous SERS detection of multiple circulating immunomarkers (soluble PD-1, PD-L1, and EGFR) present in body fluid [48]. The SERS particles developed in this report were anisotropic gold–silver alloy nanoboxes which generated stronger Raman signal intensities than typical spherical gold or silver nanoparticles. Further, the system employed nanoyeast-scFvs as the target capture agents which offered better surface functionalization. The application of this system was demonstrated for the detection of soluble PD-1, PD-L1, and EGFR in human serum at low concentrations of 6.17, 0.68, and 69.86 pg/mL, respectively [48]. 

In a separate study, Reza et al. developed a combination approach of microfluidics and SERS for the ultrasensitive detection of multiple soluble immune checkpoint biomarkers at as low as 100 fg/mL in simulated human serum samples. In this system, fluidic nanomixing within the microfluidic channels enabled the efficient capture of an extremely low amount of target immunomarker proteins. The introduction of SERS nanotags, in the format of gold nanoparticles modified with Raman reporter molecules and antibodies, as detection moieties significantly contributes to the enhanced Raman signal acquisition for the fg level detection of soluble PD-1, PD-L1, and LAG-3 within the assay platform [49].

**Figure 2:**
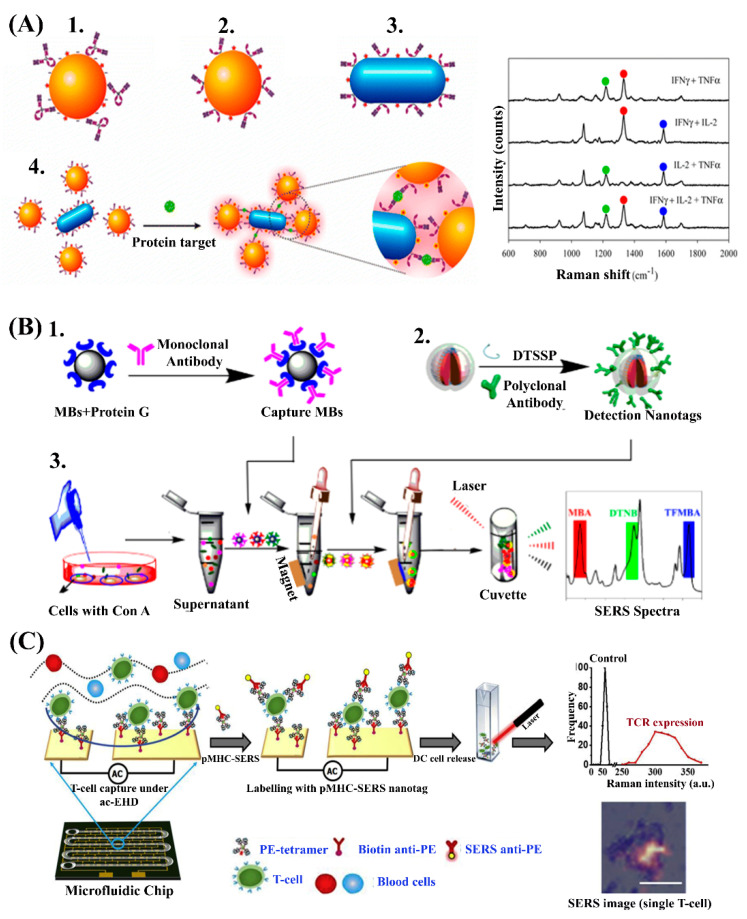
(**A**) Target-controlled assembly-based surface-enhanced Raman spectroscopy (TCA-SERS) immunoassay for multiplexed analysis of protein biomarkers. Reproduced from [50], with permission from the American Chemical Society, 2013. (**B**) SERS immunoassay for multiplexed detection of cytokines secretion. (1) Magnetic bead functionalization with capture antibody, (2) detection antibody immobilization on SERS nanotags comprising of tunable Raman reporters residing between gold cores and silver shells, and (3) schematic representation of the immunoassay. Reproduced from [52], with permission from the American Chemical Society, 2019. (**C**) An integrated SERS-microfluidic platform for antigen specific T-cell isolation and SERS mapping of T cell receptor expressions. Reproduced with permission from [53], with permission from Elsevier, 2019.

SERS-based methods have also been investigated for cytokine secretion analysis as an indicator of immune response to diseases. For example, Wang et al. reported a SERS assay, termed as target-controlled assembly-based SERS (TCA-SERS) immunoassay, for the multiplexed detection of three cytokines (IFN-γ, IL-2, and TNF-α) from complex biological samples (Figure 2A). In this study, they decorated gold nanoparticles or nanorods with antibody half-fragments, nanofluorescent Raman-active dyes, and passivating proteins which showed excellent biocompatibility and stability. This functionalization of a SERS-active nanomaterial with antibody half-fragments enabled the controlled assembly of SERS nanoparticles in response to sandwiched antibody–antigen interactions. The resultant orientation of nanoparticles in close proximities facilitated plasmonic coupling that resulted in high SERS enhancement and extremely sensitive detection of cytokines down to the pM level [50]. 

Similarly, Cao and colleagues reported a SERS sandwich immunoassay for the highly sensitive and specific detection of IL-8 from human serum samples at as low as 6.88 pg/mL [51]. In this work, they immobilized antibodies on highly branched gold nanoparticles (HGNPs)-decorated indium tin oxide glass as the capture substrates for IL-8, and utilized gold nanocages (GNCs) as SERS tags to enable the formation of sandwich structures in the presence of IL-8. The close proximities of HGNPs and GNCs during IL-8 capture resulted in the formation of interstitial regions and produced significantly enhanced Raman scattering upon laser excitation. Hence, this enabled the detection of very low concentrations of IL-8 from breast and gastric cancer patients’ serum samples.

In another study, Li et al. developed a set of SERS-active nanomaterials consisting of a gold core functionalized with Raman reporter molecules and a silver layer around the gold core. The designed orientation of gold in the center surrounded by a silver shell produces a local hotspot for Raman reporter molecules within the metal gap and contributes to a high signal intensity. Furthermore, the silver shell also protects the reporter molecules from the external environment, hence reducing any bias due to interferences. The detection methodology is straightforward via the magnetic purification of target proteins and SERS nanomaterial-labeling of target proteins before laser excitation and SERS signal acquisition. The assay is extremely sensitive and was demonstrated for the detection of multiple cytokines at as low as 4.5 pg/mL (Figure 2B) [52].

The identification and characterization of T cell subsets that play major roles in adaptive immunoresponse in the tumor microenvironment are important for delineating host immune response, new drug target selection, and therapy assessment. To enable such robust analysis, Dey et al. developed a highly sensitive integrated assay platform that leverages the benefits of microfluidics for target antigen specific T cell isolation, and SERS-active nanomaterials for the detection and characterization of T cell receptor expression heterogeneity at individual cell levels (Figure 2C) [53]. In this approach, the enhanced micromixing under an electrohydrodynamic fluid flow significantly increased the collisions between peptide–major histocompatibility complex (pMHC)-functionalized capture surface and target T cells, and contributed to the capture of 56.93 ± 7.31% of total CD4^+^ T cells in prepared samples. Furthermore, on-chip target cell staining with pMHC-SERS nanotags and subsequent retrieval of these target T cells facilitated the screening of the receptor expression level on T cells [53]. Although this method has shown significant potential for clinical application, it remains in the early phase of development and needs further validation for clinical translation. 

## 3. SERS Single Cell-Immunoimaging

Immunoimaging of multiple components of the tumor microenvironment is crucial for understanding the immunotherapy landscape, allowing the prediction of treatment outcome and monitoring of treatment efficacy [54,55]. Currently, in vitro or in vivo immunoimaging is typically achieved via immunohistochemical staining or fluorescence-based immunocytochemical imaging. However, both techniques are limited by image interpretation (i.e., pathology) expertise and variability or autofluorescence and photobleaching issues. In recent years, various new SERS-active nanomaterials have been developed to enhance performance in multiplexed SERS immunomarker imaging [40,56,57,58,59,60]. The advantages of SERS immunoimaging include high spatiotemporal resolution, multiplexing with minimal spectral overlapping, and specific Raman reporter-labeling of targets [58,61,62,63]. In recent years, there has been an increasing uptake of designed SERS-active nanomaterials for cancer immunotherapy applications, particularly for cell surface immune checkpoint biomarkers. 

**Figure 3:**
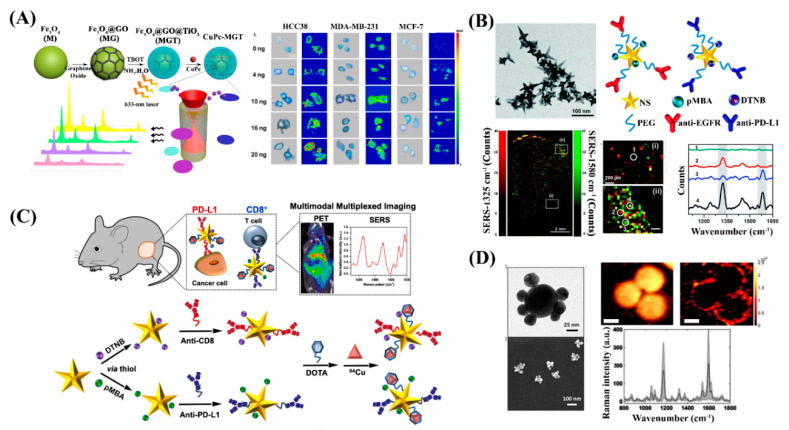
(**A**) Synthesis and enhancement mechanism of magnetic Fe_3_O_4_@GO@TiO_2_ ternary heterostructures (Left). Raman mapping images of cancer cells after different concentrations of drug treatment after 48 h (Right). Reproduced from [64], with permission from the American Association for the Advancement of Science, 2018. (**B**) Gold nanostars (AuNS) functionalized with Raman reporter molecules and antibodies for the detection of EGFR and PD-L1 (Top). Ex vivo Raman spatial maps and corresponding Raman spectra of the PD-L1 and EGFR expression using tumor tissue section (Bottom). Reproduced from [66], with permission from the Royal Society of Chemistry, 2018. (**C**) Immunoactive AuNS (IGNs) functionalized with Raman reporter molecules and anitbodies for multimodal multiplexed immunoPET-SERS imaging to detect PD-L1^+^ tumor cells and CD8^+^ T cells in tumors. Reproduced from [70], with permission from the American Chemical Society, 2020. (**D**) Au/Au core/satellite nanoparticles for immune-SERS (iSERS) microscopy of PD-L1 localization on cancer cells (Left). Fluorescence image, iSERS false-color image, and corresponding SERS spectrum (Right). Reproduced from [45], with permission from Wiley-VCH, 2020.

Tian and colleagues synthesized novel ternary heterostructure SERS nanoprobes for the evaluation of the PD-L1 expression on breast cancer cells (Figure 3A) [64]. The SERS substrate was prepared through a layer-by-layer coating process, starting with the coating of graphene oxide (GO) on iron oxide (Fe_3_O_4_) nanoparticles through electrostatic adsorption. Then, uniform porous titanium oxide (TiO_2_) shell layers were created via a versatile kinetics-controlled coating method to generate magnetic Fe_3_O_4_@GO@TiO_2_ ternary heterostructures. The synthesized ternary heterostructures were then incubated with the copper phthalocyanine (CuPc) Raman reporter and anti-PD-L1 molecules to generate the finalized plasmon-free SERS nanoprobes for the evaluation of the PD-L1 expression of cancer cell surfaces. Using these SERS nanoprobes, the enhancement factor of the CuPc SERS signal was estimated to be 8.08 × 10^6^ due to the resonance Raman effect of CuPc, charge transfer between GO and TiO_2_, and enrichment from the porous TiO_2_. Using Raman mapping imaging, the nanoprobes were successfully used for in situ quantification and imaging of the PD-L1 expression on three different triple-negative breast cancer cell lines at the single-cell level, and for monitoring the PD-L1 expression variation during IFN-γ drug treatment. 

Bardhan and co-workers also reported several progressive studies on the use of gold nanostructures for SERS imaging of checkpoint inhibition immunomarkers. They first reported a one-step HEPES (2-[4-(2-hydroxyethyl)-piperazin-1-yl] ethanesulfonic acid)-mediated synthesis of multibranched gold nanoantennas (MGNs) for the multiplexed SERS imaging of PD-L1 and EGFR with high spatial resolution [65]. The MGNs consisted of spherical cores which absorbed incident light, and multiple protrusions which routed and concentrated incident light-like nanoantennas. The sub-100 nm MGNs were then functionalized with anti-EGFR or anti-PD-L1 antibodies via a heterobifunctional linker bioconjugation chemistry and Raman molecules ρMBA and DTNB, respectively. The authors then performed multiplexed in vitro SERS imaging by simultaneously incubating MDA-MB-231 breast cancer cells with a mixture of anti PD-L1-DTNB-MGNs and anti-EGFR-ρMBA-MGNs, and achieved highly specific detection of both biomarkers. 

This initial work was then extended to using similarly shaped gold nanostars (AuNS) for both in vivo longitudinal PD-L1 and EGFR tracking, as well as ex vivo Raman mapping of whole tumor lesions (Figure 3B) [66]. Using mouse models bearing MDA-MB-231 xenografts, the researchers delivered the SERS-active nanostars via systemic injection and were able to track the AuNS longitudinally over 72 h to assess both the PD-L1 and EGFR receptor status via in vivo SERS imaging. The direct placement of a Raman fiber optic probe on the tumor xenograft for in vivo SERS measurements is ideal for subcutaneous tumors, which enables sufficient light penetration and detection of Raman scattering. This allows immunomarker monitoring during disease with minimal need for repetitive invasive biopsies. Using 5-micron tumor sections of whole tumor xenografts, the authors performed ex vivo SERS mapping of entire tissue sections and provided near cellular-level spatial and temporal resolution of the PD-L1 and EGFR expression in tumor areas. This enables the addressment of current autofluorescence and photobleaching challenges with immunofluorescence imaging. 

The development of multimodal SERS nanomaterials facilitates immunoimaging using SERS and other established imaging technologies [67,68,69]. To this end, the use of AuNS in multiplexed SERS imaging has lately been developed further into immunoactive AuNS (IGNs) for combining SERS imaging with positron emission tomography (PET) (Figure 3C) [70]. IGNs were labeled with antibodies, Raman labels, and ^64^Cu for versatile use in whole-body PET imaging and targeted immunomarker SERS imaging. Ou and colleagues demonstrated the IGNs for immunoimaging for PD-L1^+^ tumor cells and CD8^+^ T cells simultaneously with high sensitivity and specificity. Additionally, IGNs were effectively used to monitor immunotherapy responses in mice treated with a combination of anti-PD-L1 and anti-CD137 monoclonal antibodies. The extended use of this multimodal multiplexed immunoimaging method for various immunomarkers in the tumor microenvironment (inhibitory ligands such as TIM-3 or LAG-3; immune cell populations such as CD4^+^ T cells or natural killer cells) could allow for the identification of patients who will respond to immunotherapies even before treatment commencement.

Schlücker and colleagues employed Au/Au core/satellite nanoparticles as SERS nanotags for immune-SERS microscopy (iSERS) of PD-L1 localization on single breast cancer cells (Figure 3D) [45]. The SERS nanotags exhibited remarkable signal brightness and colloidal stability under laser excitation and iSERS has the main benefit of significantly reducting cell autofluorescence due to the use of red to near-infrared laser excitation. The SERS nanotags were synthesized by first coating positively charged 50 nm Au nanosphere cores with the MMC (7-mercapto-4-methylcoumarin) Raman reporter and negatively charged 30 nm Au nanoparticle satellites. The Au/Au core/satellite nanoparticles were then conjugated to anti-PD-L1 antibodies via EDC (1-Ethyl-3-(3-dimethylaminopropyl) carbodiimide)/NHS (*N*-hydroxysuccinimide) chemistry. Using the synthesized SERS nanotags in iSERS, the authors demonstrated the capability for the specific imaging of the PD-L1 expression on single SkBr-3 cells.

## 4. SERS Immunotherapeutic Drug Screening and Delivery

SERS-active nanomaterials can be imbued with compelling traits for drug screening and delivery applications [67,71,72,73,74,75]. This extended the potential translational applications of SERS from biomarker sensing/imaging (as discussed in prior sections) to drug screening/delivery in the process of cancer immunotherapy. Explicitly, the multiplexing capability and high signal enhancement effect of SERS in small volume reactions of SERS is fitting for high-throughput screening for therapeutic candidates, and the photon excitation mechanism of SERS is also optimal for administrating photothermal therapy to kill targeted cancer cells. 

**Figure 4:**
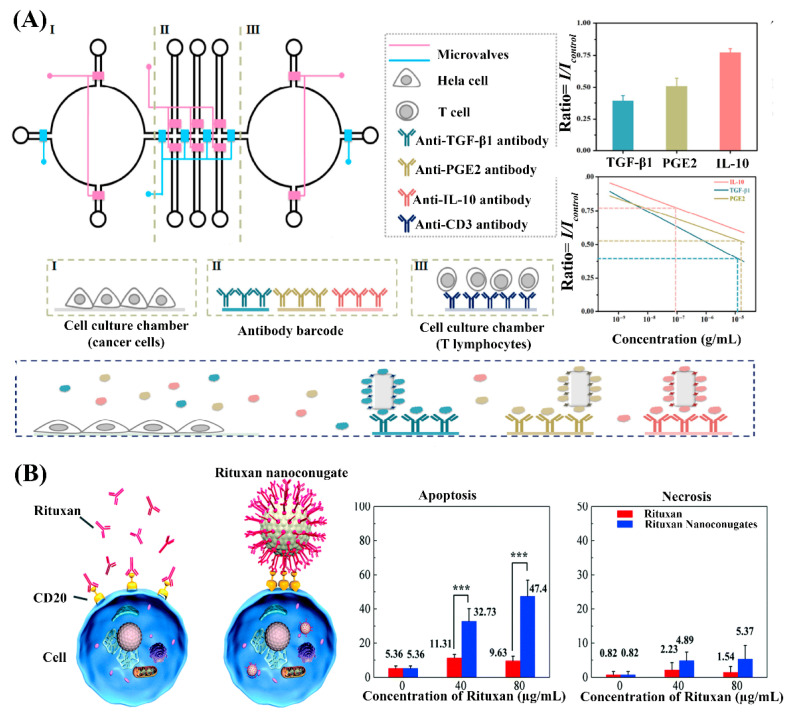
(**A**) Quantitative and multiplexed screening of immunosuppressive proteins (TGF-β1, PGE2, and IL-10) by a combination of SERS nanoprobes with microfluidic networks. Reproduced from [76], with permission from Springer, 2017. (**B**) Enhanced capping mechanism of CD20 molecules by nanoconjugation of Rituxan to SERS-active silver nanoparticles (Left). Rituxan nanoconjugates induce more apoptosis and necrosis in lymphoma cells than unconjugated Rituxan (Right). Reproduced from [77], with permission from the Royal Society of Chemistry, 2017.

Wu and co-workers reported the combination of SERS nanoprobes and microfluidics that gave rise to a fully integrated and highly automated platform for probing intercellular communications in a tumor microenvironment and testing potential drug candidates (Figure 4A) [76]. Using SERS-active nanomaterials in the form of antibody-labeled gold@silver core–shell nanorods, the secretion of immunosuppressive proteins (TGF-β1, PGE2, and IL-10) was characterized through multiplexed detection with high 1 ng/mL sensitivity. The microfluidic compartments provided the conditions to culture both human cervix carcinoma (HeLa) cells and T cells side by side and facilitate the simulation of cancer cell–immune system communications. Furthermore, on-chip screening of anti-cancer drug (artesunate) response was demonstrated through the quantitative detection of SERS signals of respective immunosuppressive proteins secreted into the extracellular environment by cancer cells. Through realistic expansion of throughput capability on the microfluidic platform, SERS-active nanomaterials could ideally be further harnessed for the screening of different immunotherapeutic drugs. 

In a separate approach, Yao et al. demonstrated the potential of the dual functionality of SERS nanomaterials for lymphoma cell detection as low as single cell and the enhancement of immmunotherapy efficiency (Figure 4B) [77]. In this study, the authors reported a method for preparing Rituxan (monoclonal antibody drug for lymphoma)-functionalized 50 nm silver nanoparticles to label and detect lymphoma cells. The silver nanoparticles were prior coated with a layer of ρMBA Raman reporter molecules through thiol molecules before covalent conjugation of Rituxuan using EDC-NHS coupling. Rituxan targets the CD20 immunomarker on malignant cells, and the resident time on the cell surface is important for the Rituxan and CD20 interaction, as well as drug internalization and cell killing. The nanoconjugation of Rituxan to SERS-active silver nanoparticles was shown to enhance a cap formation of CD20 clustering on the cell membrane, and increased drug efficiency by ~21% for inducing cell death as compared with unconjugated Rituxan treatment. This nanoconjugate construct is a promising tool in lymphoma theranostics by integrating the specific SERS detection of CD20(+) lymphomas cells with enhanced drug therapy at the molecular level. 

SERS-active nanomaterials can serve as an externally controlled light-triggered therapeutic tool upon the specific detection of cancer immunomarkers. Webb and colleagues utilized antibody-labeled MGNs to target PD-L1 and EGFR on cell surfaces and enabled receptor-specific photothermal therapy (PTT) using an 808 nm laser for 15 min at 4.7 W/cm^2^. PTT was performed on both breast cancer MDA-MB-231 and MCF7 cell lines to demonstrate minimal non-specific binding and off-site toxicities, as well as highly specific and sensitive spatiotemporal cell death control with low laser power. This methodology shows the potential of theranostic SERS-active nanomaterials for predictive and personalized cancer immunotherapy via image-guided immunomarker imaging and PTT.

## 5. Outlook

As discussed in the preceding sections and ongoing research studies, the versatility of SERS-active nanomaterials for different biomarkers has been well demonstrated. From a manufacturing standpoint, the mass production of basic SERS-active nanomaterials in the form of gold or silver nanoparticles is economically feasible and commercially available at present. Although SERS-active nanomaterials have offered opportunities in the use of SERS for immunomarker profiling within the tumor microenvironment, there remain nanomaterial-related challenges to be resolved in both technical (better SERS detection performance) and biological (providing clinically useful information) contexts. As ascertained advances into biological mechanisms, biomarker developments, and novel drug designs of cancer immunotherapies continue into the foreseeable future, efforts should similarly be made to progress SERS-active nanomaterials towards clinical translation and commercialization in immunotherapeutic applications.

**Figure 5:**
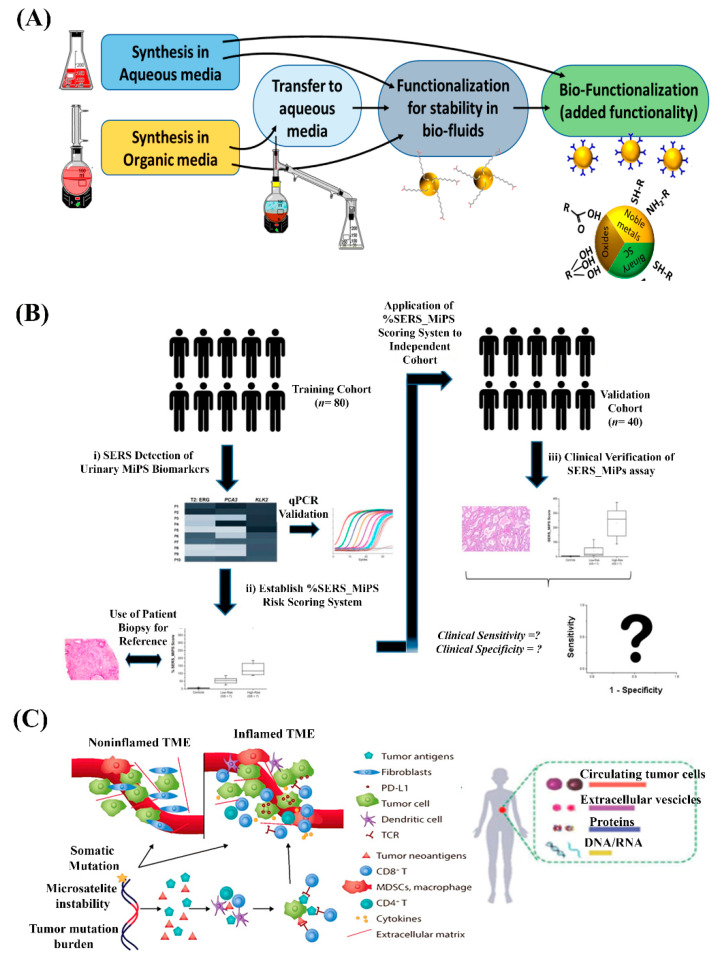
(**A**) Approaches for the stabilization of SERS-active nanomaterials in biofluids using functionalization of various surface ligands. Reproduced from [78], with permission from MDPI, 2018. (**B**) Clinical validation blueprint for the translation of SERS nanomaterials using a proven biomarker panel and independent training and validation patient sample cohorts. Reproduced from [81], with permission from the American Chemical Society, 2018. (**C**) The expansion of SERS-active nanomaterial usage for an entire repertoire of molecular immunomarker species (cells, DNA, RNA, proteins, etc.) in the tumor microenvironment. Reproduced from [16], with permission from the American Association for Cancer Research, 2018.

One crucial step forward is the investigation of stability and interactions of SERS-active nanomaterials in the tumor microenvironment, especially for in vivo applications. Firstly, the stability of SERS-active nanomaterials in biological fluids is associated with rational nanoscale design and fabrication criteria for biocompatibility (Figure 5A) [78,79]. Thence, considerations into surface modification strategies of SERS-active nanomaterials should be taken into consideration to prevent aggregation and minimize surface fouling from the rich excess of non-target biomolecules in the tumor microenvironment. Secondly, the interactions of nanomaterials with biological molecules can cause unintended immune responses such as inflammation or apoptosis. Thus, an in-depth understanding into the mechanisms and immune consequences of SERS-active nanomaterials within the tumor microenvironment is important for safe and accurate use in different cancer immunotherapy applications. 

Next, extensive clinical validation of SERS-active nanomaterials for immunomarker sensing, imaging, and drug delivery should be performed to quantitatively measure clinical performance beyond proof-of-principle analytical studies [80]. To evaluate SERS nanomaterials clinically, an archetypical approach as outlined in our recent research study may be employed (Figure 5B) [81]. In concise terms, our clinical validation blueprint involves the identification and selection of a clinically proven biomarker model for testing, as well as use of independent training and validation patient sample cohorts to minimize the evaluation bias. Optimistically, this can further convince the feasibility of a novel SERS-active nanomaterial for patient benefit and progress beyond academic research into clinical settings.

Lastly, we are increasingly observing the integration of multiple molecular species (e.g., DNA, RNA, proteins) as a single cancer biomarker signature to provide a more comprehensive profile of tumor biology as compared with the use of a single molecular species. As the current research endeavors reviewed herein are mainly focused on protein-based immunoassays, it is foreseen that the use of SERS-active nanomaterials can quintessentially be extended to nucleic acid biomarkers for cancer immunotherapy applications [82,83,84]. Given the biological complexity of the molecular landscape within the tumor microenvironment, overcoming the challenge in expanding the application repertoire of SERS-active nanomaterials for multiple molecular species would fully utilize the versatility, sensitivity, and multiplexibility of SERS (Figure 5C). 

## 6. Conclusions

The vast possibilities of SERS-active nanomaterials have enabled the modern venture into various application domains of cancer immunotherapies. Such nanomaterial strategies have taken decades to attain the present progress of SERS in the biomedical field, and the works which have been reviewed herein indicate the tantalizing prospects of extending the unique characteristics of SERS for the sensing, imaging, and drug treatment of cancer immunomarkers in the complex tumor microenvironment.
